# National Association of Dental Faculties

**Published:** 1899-09

**Authors:** F. Hise


### PROCEEDINGS.

### National Association of Dental Faculties.

REPORTED BY F. HISE.

The sixteenth annual session of the National Association of Dental Faculties was held in Niagara Falls, commencing Friday, July 28, 1899.

The following colleges were represented.

Birmingham Dental College, Birmingham, Ala.—T. M. Allen.

University of California, Dental Department, San Francisco, Cal.—A. A. d'Ancona.

Colorado College of Dental Surgery, Denver, Col.—J. S. Jackson.

University of Denver, Dental Department, Denver, Col.—A. H. Sawins.

Columbian University, Dental Department, Washington, D. C.—J. R. Hagan.

Howard University, Dental Department, Washington, D. C. —A. J. Brown.

National University, Dental Department, Washington, D. C. —A. D. Cobey.

Atlanta Dental College, Atlanta, Ga.—H. R. Jewett.

Dental Department of Atlanta College of Physicians and Surgeons, Atlanta, Ga.—Frank Holland, S. W. Foster.

Chicago College of Dental Surgery, Chicago, Ill.—Truman W. Brophy.

Northwestern University Dental School, Chicago, Ill.—Theo. Menges.

Indiana Dental College, Indianapolis, Ind.—George E. Hunt.

State University of Iowa, Dental Department, Iowa City, Ia.—W. S. Hosford.

Louisville College of Dentistry, Louisville, Ky.—H. B. Tileston.

Baltimore College of Dental Surgery, Baltimore, Md.—M. W. Foster.

University of Maryland, Dental Department, Baltimore, Md. —John C. Uhler.

Boston Dental College (Tufts College Dental School), Boston, Mass.—Chas. P. Thayer.

Harvard University, Dental Department, Boston, Mass.—Thomas Fillebrown.

College of Dental Surgery of the University of Michigan, Ann Arbor, Mich.—J. Taft, N. S. Hoff, alternate.

Detroit College of Medicine, Dental Department, Detroit, Mich.—G. S. Shattuck.

University of Minnesota, Dental Department, Minneapolis, Minn.—W. P. Dickinson.

Kansas City Dental College, Kansas City, Mo.—J. D. Patterson.

Western Dental College, Kansas City, Mo.—D. J. McMillan.

Marion-Sims College of Medicine, Dental Department, St. Louis, Mo.—J. H. Kennerly.

Missouri Dental College, St. Louis, Mo.—A. H. Fuller.

University of Omaha, Dental Department, Omaha, Neb.—A. O. Hunt.

University of Buffalo, Dental Department, Buffalo, N. Y.—William C. Barrett, R. H. Hofheinz.

New York College of Dentistry, New York City—Faneuil D. Weisse.

New York Dental School, New York City—John I. Hart, Roderick M. Sanger.

Cincinnati College of Dental Surgery, Cincinnati, O.—G. S. Junkerman, W. T. McLean.

Ohio College of Dental Surgery, Cincinnati, O.—H. A. Smith.

Western Reserve University, Dental Department, Cleveland, O.—H. L. Ambler.

Ohio Medical University, Dental Department, Columbus, O. —Otto Arnold.

Pennsylvania College of Dental Surgery, Philadelphia, Pa.—Wilbur F. Litch.

Philadelphia Dental College, Philadelphia, Pa.—S. H. Guilford.

University of Pennsylvania, Dental Department, Philadelphia, Pa.—James Truman, Edward C. Kirk.

Pittsburg Dental College, Pittsburg, Pa.—Walter H. Fundenburg.

School of Dentistry, Central Tennessee College, Nashville, Tenn.—G. W. Hubbard.

University of Tennessee, Dental Department, Nashville, Tenn.—L. G. Noel.

Vanderbilt University, Dental Department, Nashville, Tenn.—Henry W. Morgan.

Tacoma College of Dental Surgery (North Pacific Dental College), Portland, Ore.—Geo. H. Chance.

Milwaukee Medical College, Dental Department, Milwaukee, Wis.—Geo. V. I. Brown.

Royal College of Dental Surgeons of Ontario, Toronto, Canada—J. B. Willmott.

The treasurer reported that the dental department of Tennessee Medical College, of Knoxville, Tenn., was no longer in existence, having been absorbed by another school.

The Tacoma College of Dental Surgery, having removed to Portland, Ore., was given authority to change its name to North Pacific Dental College.

The trustees of Boston Dental College accredited Dr. C. P. Thayer as delegate to explain to the Association that they had transferred the institution, with all its appurtenances, to Tufts College, and to request that the Tufts College Dental School be permitted to make application for membership at this meeting. On motion it was ordered that Tufts College Dental School be accepted as a continuance of the old college and that the change of name be approved.

The applications for membership of the following schools, having been reported as regular by the Executive Committee, lie over for one year for final action :

Medico-Chirurgical College of Philadelphia, Dental Department, Philadelphia, Pa.; Central College of Dentistry, Indianapolis, Ind.; College of Dentistry, University of Southern California, Los Angeles, Cal.; Illinois School of Dentistry, Chicago, Ill.; Washington Dental College and Hospital of Oral Surgery, Washington, D. C.; Keokuk Medical College, Dental Department, Keokuk, Ia.

The Committee on Text-Books reported recommending that the following be adopted: "Anatomy and Histology of the Mouth and Teeth," by I. N. Broomell, D.D.S.; "The Practice of Dental Medicine," by Geo. F. Eames, M.D., D.D.S.; "Comparative Dental Anatomy," by A. H. Thompson, D.D.S. (recommended last year in proof); "Methods of Filling Teeth," second edition, by R. Ottolengui, M.D.S.

The committee had also examined "Chemistry and Metallurgy Applied to Dentistry," by Vernon J. Hall, Ph.D., and, while admirable and containing many excellent features, the committee believed it unwise to recommend it as a text-book, inasmuch as there are already two excellent works on the same subject on the list.

Of "Interstitial Gingivitis, or so-called Pyorrhea Alveolaris," by Eugene S. Talbot, M.D., D.D.S., the committee reported that it contained evidence of laudable and extensive research, but the subject is still a matter of so much controversy and diversity of opinion as to make undesirable a text-book upon it at the present time.

The committee also suggested the removal of Clifford's "Manual of Recitations," adopted in 1892, and Burchard's "Compend of Pathology," adopted in 1897.

The following resolutions, laid over under the rules from 1898, were adopted:

Offered by Dr. Allen:

*Resolved*, That it is the sense of this association that the present method of bestowing scholarships is no longer called for, and is detrimental to the best interests of the profession, and that hereafter no college of this association shall grant either free or beneficiary scholarships not absolutely made obligatory in their charter.

Offered by Dr. Barrett:

*Resolved*, That it shall be the duty of the secretary of this association to present at the opening of each annual session a list of the colleges, members of this association, which have been unrepresented for two years, that proper action may be promptly taken.

The resolutions of Drs. Allen and d'Ancona, concerning the attendance of students, were substituted by the following, offered by Dr. Willmott, which was adopted:

*Resolved*, That students in attendance at colleges of this association, to obtain credit for a full term, must be and remain in attendance until the close of the session.

In accordance with this action Rule 4 was amended to read as follows:

4. In cases where a regularly matriculated student, on account of illness, financial conditions, or other sufficient cause, abandons his studies for a time, he may re-enter his college at the same or a subsequent session, or where, under similar circumstances, he may desire to enter another college, then, with the consent of both deans, he may be transferred.

Rule 9 was amended to read as follows:

Admission of Undergraduates of Medicine.

9. Undergraduates of reputable medical colleges who have regularly completed one full scholastic year of a six months' term, and passed a satisfactory examination in the studies of the freshman year, may be admitted to the junior grade in colleges of this association, subject to other rules governing admission to that grade.

The Committee on Conference with the National Association of Dental Examiners reported, as the result of several conferences held with a similar committee from the Examiner's Association, that an agreement had been reached concerning the matters which had been in controversy between the two associations for several years. The report was adopted. [The basis of the agreement, with some account of the difficulties referred to, will be found at the end of this report.]

The following resolution was unanimously adopted:

*Resolved*, That the thanks of the National Association of Dental Faculties are due to the Chicago College of Dental Surgery for the courage and persistence with which it has maintained what we believe to be a correct principle, and that we regard the placing as unrecognized and disreputable in the newspapers and otherwise of one of the oldest and best of our professional teaching institutions an injustice that demands complete rectification.

Dr. Barrett offered the following, which were adopted:

*Resolved*, That the commonly accepted Code of Ethics regulating the conduct of practitioners in their relations with other practitioners be approved and made obligatory upon the dental colleges of this association in their relations with other colleges.

*Resolved*, That the section of the Code which refers to public advertisements be interpreted to forbid the advertising of the infirmaries of dental colleges in any manner that might be construed to be unprofessional if done by a practitioner.

*Resolved*, That as dental colleges should in every practicable manner impress the importance of ethical conduct upon their students, and should themselves set a good example in this particular, their public advertisements should be confined to a simple statement of the location of the schools, the date of opening and closing, with any other really essential facts, all details being reserved for the annual announcement, which itself shall not violate the usually accepted ethical tone.

Dr. Taft offered the following:

*Resolved*, That a commission, consisting of three persons, be appointed, whose duty it shall be to take cognizance of, investigate, and advise with any parties contemplating the establishment of a new college or the reorganization of an old one.

In the performance of the duties of this commission it shall be competent to take into consideration the following points, viz:

The consideration of any proposed new dental college, taking into account all the circumstances that attach to it, the motive that prompts such an organization, the need for it, the proposed locality, the character and ability of those who propose to conduct it, the sufficiency of the resources that may be available for its establishment, and whether on the part of the promoters there is a just appreciation of that which is required for such an institution.

The attainment of full knowledge on these points would enable the commission to advise wisely.

It will be the duty of this commission to report to this body at each annual meeting.

The resolution was adopted, and it was ordered that the commission be elected with the other officers.

The following amendment to the constitution was adopted:

Change Article V to read as follows:

Article V. The Executive Committee shall consist of five members, three of whom shall be elected annually; the two receiving the higher number of votes shall hold office for two years each. The Executive Committee shall have power to designate the time and place of meeting, make preparations for same, and transact such other business as usually devolves upon such committee. That five members be elected this session, the two receiving the higher number of votes to serve for two years, the other three for one year each.

On motion of the Executive Committee it was ordered that colleges making application for membership in this body shall have present a copy of their annual announcement, and that a duly authenticated representative of the school be present at the meeting, without which the application shall not be considered.

It was decided that the change from six to seven months' terms, which goes into effect with the session of 1899-1900, should apply to all students in colleges of the association, even though the students may have previously attended under the six months' rule.

On motion of Dr. Barrett it was ordered that a Committee on Law, to consist of three members, be elected to serve as a standing committee, which shall be authorized to levy such assessments upon the members of the association as may be necessary for the payment of past legal expenses and such as may accrue in the future in the suppression of the issue of fraudulent diplomas. Such assessments to be lodged with the treasurer and paid upon the order of the Committee on Law.

It was also ordered that all legal matters which may arise in connection with the National Association of Dental Faculties shall be referred to this committee.

The Committee on Foreign Relations, in concluding the report of its work for the year, offered the following resolutions, which were adopted:

*Resolved*, That the Foreign Relations Committee be instructed to take any steps which they may deem advisable for the putting an end to the issuing of fraudulent and irregular degrees, and to this end are authorized during the coming year to use any funds in the treasury of the association upon the approval of the Law Committee.

*Resolved*, That the European Advisory Board of the Foreign Relations Committee be and is hereby invited each year to send a delegation to attend the annual meeting of this association, and that such delegation be accorded seats in the meetings of the association, with all the privileges of debate.

*Resolved*, That no student coming from Europe shall be received by any member of the association until his credentials shall have been approved by the members of the European Advisory Board for the country from which he claims to have come.

*Resolved*, That the Committee on Foreign Relations be authorized to appoint advisory boards for countries outside of Europe, whenever in their judgment it is advisable to do so, and report any such action at the next succeeding meeting of this association.

*Resolved*, That the Foreign Relations Committee be given jurisdiction in all foreign American dental educational matters, subject always to the approval of the National Association of Dental Faculties, to which a full written report shall be submitted annually.

Following are the members of the European Advisory Board, so far as appointed:

Great Britain—Wm. Mitchell, W. E. Royce, B. J. Bonnell.

Holland and Belgium—J. E. Grevers, Ed. Rosenthal, C. van de Hoeven.

Denmark, Norway and Sweden—Elof Forberg.

Germany—W. D. Miller, C. F. W. Bodecker, —— Hesse.

Italy and Greece—Albert T. Webb, Tullio Avanzi, A. V. Elliott.

France—J. H. Spaulding, I. B. Davenport, G. A. Roussel.

Spain and Portugal—Florestan Aguilar, —— Purtuondo, —— Thomas.

Switzerland and Turkey—L. C. Bryan, Theo. Frick, Paul Guye.

Japan, China and Corea—Louis Ottofy.

Australia and New Zealand—Alfred Burne.

The following resolution, offered last year, was again laid over for another year:

Offered by Dr. Hosford:

*Resolved,* That a four years' course in a reputable college, leading to the degree of A.B., Ph.B., or B.S., or four years of biological work, be accepted as one year's credit in the colleges of this association, subject to other rules governing admission to second year grade.

*Resolved,* That students matriculated in both a collegiate and dental department of a university, having completed the work of the first year in dentistry during the four year collegiate course, may, on graduation with collegiate degree, be given full credit for one year in colleges of this association.

The following, offered by Dr. Foster, was referred to the Executive Committee to be reported upon next year:

*Resolved,* That when a student fails in any part of the requirements for obtaining his final degree, such student must hold over till the next regular course, during which time he may re-enter and remove such conditions by completing his work and can only apply for his degree at the close of term as announced in the catalogue of such school.

The following resolutions lie over under the rules till next year:

Offered by Dr. Barrett:

To change Rule 1 to read as follows:

PRELIMINARY EXAMINATIONS.

1. The following preliminary examination shall be required of students seeking admission to colleges of this association:

*a.* The minimum preliminary educational requirement of colleges of this association, after the session of 1901–1902, shall be a certificate of entrance into the third year of a high school, or its equivalent, the preliminary examination to be placed in the hands of the State Superintendent of Public Instruction.

*b.* Nothing in this rule shall be construed to interfere with colleges of this association that are able to maintain a higher standard of preliminary education.

Offered by Dr. Weisse:

*Resolved,* That Rules 8, 9, and 10 of the Code of Rules be rescinded, and the following be substituted therefor:

That advanced standing to the junior or senior classes of institutions of this association shall only be upon certificate of one or two sessions' attendance, respectively, in an institution belonging to this association.

Offered by Dr. Truman:

*Resolved,* That members of this association violating the rules of this body shall, upon conviction, be fined not less than one hundred dollars for each offense, or be subject to censure, suspension, or expulsion, at the pleasure of the association.

Offered by Dr. Barrett:

*Resolved,* That the Executive Committee be instructed that, except under what they shall decide to be unusual or extraordinary circumstances, and which in their report they shall detail to the association, they shall not report favorably any application for the admission of a new college in the following instances:

1. When there has not been actually secured and bought, or leased for a term of not less than three years, and fitted up with all required equipments, a sufficiently commodious and convenient building, entirely adequate to the needs of not less than one hundred students. Such equipment shall include not only the laboratories, infirmaries, etc., with proper chairs, benches, and all apparatus required for complete practical dental instruction, but the rooms and fittings necessary for scientific training, with apparatus and equipments necessary for the proper teaching of bacteriology, histology, microscopy, chemistry, and such other cientific studies as should form a part of an advanced curriculum of study.

2. When the character and attainments of its faculty, which must already have been named, and a list of the members of which, with the respective positions they are to occupy, shall be embodied in the application presented, are not such as to give assurance that the school will be conducted in a manner to reflect credit upon the dental profession and to insure complete and adequate instruction in all branches of a broad dental curriculum of study.

3. When the proposed dental college or department is evidently and unmistakably intended primarily for the purpose of sustaining or strengthening another existing institution with which it is to be allied.

4. When the city or town in which such college is to be located already contains a college, or colleges, for dental teaching, of acknowledged efficiency, liberal character, and ethical standing, sufficient in their opinion for the promotion of the best interests of dentistry and the dental profession.

Offered by Dr. Guilford:

*Resolved*, That while examinations for progress should continue to be held annually upon the subjects taught during the year, no final examinations shall be held until the close of the third year.

Dr. Taft, from the Committee on Curriculum, submitted as the report of his committee the following:

SCHEDULE OF STUDIES.

| FIRST YEAR. | Hrs. Per Wk. | SECOND YEAR. | Hrs. Per Wk. | THIRD YEAR. | Hrs. Per Wk. |
|---|---|---|---|---|---|
| Anatomy and Dissection | 2 | Anatomy, regional | 1 | Therapeutics | 1 |
| Physiology | 2 | " comparative | 1 | Pathology | 1 |
| Chemistry, inorganic | 2 | Physiology | 2 | Surgery, general | 1 |
| " laboratory | 4 | Chemistry, organic | 2 | " oral | 1 |
| Dental Anatomy | 2 | " laboratory | 4 | Jurisprudence | 1 |
| Prosthetic Technic | 10 | Metallurgy, didactic | 1 | Orthodontia, didactic | 1 |
| Histology, didactic | 4 | " laboratory | 2 | " practical | 1 |
| " laboratory | 4 | Materia Medica | 1 | Operative Dentistry | 2 |
| Materia Medica | 3 | Operative Technic | 4 | Prosthetic Dentistry | 2 |
| Comparative Anatomy | 2 | Bacteriology, didactic | 4 | Electricity | — |
| | | Operative Dentistry, didactic | 2 | Ethics | — |
| | | Orthodontia Technic | 1 | History | — |
| | | Pathology | 2 | | |
| | | Orthodontia, didactic | 2 | | |
| | | INFIRMARY. | | | |
| | | Prosthetic Dentistry | 5 | Prosthetic Dentistry | 6 |
| | | Crown and bridge-work | 3 | Operative Dentistry | 15 |
| | | | | Crown and bridge-work | 4 |

The following were elected officers for the ensuing year: Jonathan Taft, President; B. Holly Smith, Vice-President; J. H. Kennerly, Secretary; Henry W. Morgan, Treasurer; S. W. Foster, J. B. Willmott, Executive Committee for two years; H. B. Tileston, Theo. Menges (chairman), S. H. Guilford, Executive Committee for one year; W. T. McLean, J. D. Patterson, W. S. Hosford, *Ad interim* Committee; Truman W. Brophy, Edward C. Kirk, Albert H. Fuller, Commission on Proposed New Colleges; A. O. Hunt, Henry W. Morgan, W. C. Barrett, Committee on Law.

The newly-elected President appointed the following committees: T. M. Allen, W. S. Hosford, W. P. Dickinson, G. S. Shattuck, J. G. Templeton, Committee on Schools; A. J. Brown, John I. Hart, Thomas E. Weeks, Edward C. Kirk, Thomas Fillebrown, Committee on Text-Books; W. C. Barrett, J. D. Patterson, T. W. Brophy, S. H. Guilford, H. W. Morgan, Committee on Foreign Relations; N. S. Hoff, G. V. I. Brown, Committee to Secure Papers to be Read at the Next Annual Meeting; S. H. Guilford, W. F. Litch, N. S. Hoff, A. H. Fuller, C. L. Goddard, Committee on Curriculum.

The Executive Committee reported that it had decided to adopt the suggestion of Dr. Willmott to convene the next meeting on the day of the adjournment of the National Dental Association, at the same place.

Adjourned to meet at Old Point Comfort, Friday, June 29, 1900.

An important fact in connection with the meeting of the National Association of Dental Faculties was the presence of three of the members of the European Advisory Board of the Committee on Foreign Relations: Drs. Lyman C. Bryan, of Basel, Switzerland; John E. Grevers, of Amsterdam, Netherlands, and William Mitchell, of London, England.

Dr. Grevers, in speaking of the reception to advanced standing of students from foreign countries, probably struck the keynote of the entire situation. He was impressed, he said, with the idea that the foreigner comes to this country to study dentistry for one of two reasons: First, as a graduate, or as one having fulfilled the requirements in his own country, who desires to still further develop his manipulative ability by the acquirement of American methods; or, second, because he can not fulfill the requirements in his own country and hopes to secure something here which will enable him to return home and practice. So that if the applicant from a European country is not supplied with the proper certificates the colleges should be cautious about receiving him to advanced standing.

The proceedings of the late meeting were varied by two pleasant, albeit unusual, incidents.

The first of these was a trolley ride of the members of the association and their friends to Buffalo, twenty-five miles away, and return, as the guests of the Dental Department of the University of Buffalo. Arrived at Buffalo, they were taken to the college building, where an ample collation was served, accompanied by several felicitous speeches. The various departments of the college were then inspected and pronounced good, after which the party again boarded the trolley cars and were taken to view the grounds where the Pan-American Exposition is to be held two years hence. Then came the return to Niagara Falls, which was accomplished without incident and without fatigue, every one expressing his gratification over the outing.

The second was of the same nature, but involved a visit to a foreign land. The Royal College of Dental Surgeons of Ontario invited the members of the Faculties Association and also those of the National Association of Dental Examiners to visit the college and view the city of Toronto. In response about seventy-five persons took the train at Niagara Falls for Lewiston, where they boarded the steamer for the journey across Lake Ontario to Toronto. Arrived here, a short walk brought them to McConkey's, where a fine collation was served and appropriately disposed of. Tally-hos and carriages then conveyed the party to various points of interest in the city, among others Parliament House, where they alighted and spent a short time admiring its beauty of architecture and internal arrangement and fittings. A short drive brought them to the Royal College of Dental Surgeons of Ontario, where they were assembled in the main lecture room, and speeches of felicitation and good will followed, after which the visitors circulated through the building, inspecting the equipment of the college and having explained to them the methods of instruction in various branches. It was the universal opinion that the school was admirably equipped for the systematic instruction of students of dentistry. The entrance to the college was tastefully draped with the flags of Great Britain and the United States. From the college the party proceeded to the Foresters' Temple Cafe, where a second collation was served, after which they were driven to the steamboat landing. As the vessel moved off three cheers for the Royal College of Surgeons were given with a will. The return journey was made without mishap, and the excursionists unanimously declared they had one of the most enjoyable outings of their lives.

The members of the dental profession will be glad to learn that the differences existing for some years between the National Association of Dental Faculties and the National Association of Dental Examiners have been reconciled. These differences have been the cause of much friction between the two bodies.

The cause of the trouble was the refusal of the colleges to accept various rules which have crystallized into what is known as Rule 8 of the code of Rules, Sections 1 and 2, of the Examiners' Association, because the colleges were not consulted in its framing.

The attempted enforcement of this rule recently led to litigation in the State of Wisconsin. The State Board of Dental Examiners of that State refused to admit to registration the diplomas of the Chicago College of Dental Surgery, the Northwestern University Dental School, the Pennsylvania College of Dental Surgery, the Ohio Medical University Dental Department, the Philadelphia Dental College, and others, on the ground that they did not in their preliminary examination come up to the standard established by Rule 8, and demanded that graduates of these institutions presenting diplomas for registration should submit to examination by the board as to their qualifications to practice dentistry.

This contention of the board was resisted by a graduate of the Chicago College of Dental Surgery, who brought mandamus proceedings to compel the board to accept his diploma. The board moved to quash the proceedings, which motion was denied by the court, with leave to the board to file its answer. The answer was filed, and the case was in that condition at the time of the meeting of the two associations at Niagara Falls on the 28th of July, 1899.

With a view to the adjustment of the difficulty committees of conference were appointed by the two bodies, which, after going over the matters in dispute, agreed on the side of the National Association of Dental Examiners to recommend that Rule 8 be rescinded; that all colleges having membership in the National Association of Dental Faculties be placed upon the list of recognized schools, and that all litigation be withdrawn; and on the side of the National Association of Dental Faculties that a new rule governing the preliminary requirements for admission to the college courses should be adopted.

This action was ratified by the associations. The Examiners' Association adopted a new Rule 8, Sections 1 and 2 of which read as below, the remainder of the rule being substantially as before:

Rule 8, new Sections 1 and 2:

"Section 1. Colleges desiring recommendation to the State boards by the National Association of Dental Examiners shall make application for such recommendation, through the Committee on Colleges, on blanks provided for that purpose. This rule to apply only to schools making application to the National Association of Dental Examiners for recommendation and such schools as may be dropped.

"Section 2. The following preliminary examination shall be required of students seeking admission to colleges recommended by this association. The minimum preliminary educational requirements of colleges of this association for the session of 1899–1900 shall be a certificate of entrance into the second year of a high school or its equivalent, the preliminary examination to be placed in the hands of the State Superintendent of Public Instruction, as adopted by the State Board of Missouri."

The Faculties' Association adopted the following rule governing the preliminary educational requirements of students:

"The minimum preliminary educational requirement of colleges of this association for the session of 1899-1900 shall be a certificate of entrance into the second year of a high school or its equivalent, the preliminary examination to be placed in the hands of the State Superintendent of Public Instruction.

"Nothing in this rule shall be construed to interfere with colleges of this association that are able to maintain a higher standard of preliminary education."

The cause of friction being removed, the disputes which have arisen, there is every assurance will be speedily adjusted, and the two bodies will hereafter work in harmony.

